# Comparing Item Performance on Three- Versus Four-Option Multiple Choice Questions in a Veterinary Toxicology Course

**DOI:** 10.3390/vetsci5020055

**Published:** 2018-06-09

**Authors:** Kenneth Royal, David Dorman

**Affiliations:** 1 Department of Clinical Sciences, North Carolina State University, Raleigh, NC 27695, USA; 2 Department of Molecular and Biomedical Sciences, North Carolina State University, Raleigh, NC 27695, USA

**Keywords:** assessment, medical education, veterinary medicine, testing, multiple-choice questions, psychology, toxicology, measurement, item writing, veterinary education

## Abstract

Background: The number of answer options is an important element of multiple-choice questions (MCQs). Many MCQs contain four or more options despite the limited literature suggesting that there is little to no benefit beyond three options. The purpose of this study was to evaluate item performance on 3-option versus 4-option MCQs used in a core curriculum course in veterinary toxicology at a large veterinary medical school in the United States. Methods: A quasi-experimental, crossover design was used in which students in each class were randomly assigned to take one of two versions (A or B) of two major exams. Results: Both the 3-option and 4-option MCQs resulted in similar psychometric properties. Conclusion: The findings of our study support earlier research in other medical disciplines and settings that likewise concluded there was no significant change in the psychometric properties of three option MCQs when compared to the traditional MCQs with four or more options.

## 1. Introduction

Knowledge about a subject area remains an important framework for evaluating progress in the medical profession [1]. Multiple choice questions (MCQs) are widely used in medical curricula to assess the acquisition of factual knowledge in a subject area [2,3]. Other attractions to using MCQ format exams include the ability of an examiner to test large numbers of students with minimal resources, ease of grading including the use of non-expert and machine graders, high content coverage, and the ability to obtain highly reliable and accurate scores [2]. The use of MCQs, however, does have several important drawbacks since they often do not assess higher order thinking and may lack authenticity [2,3].

One defining feature of MCQs is the presence of two or more answer options. One challenge educators face in creating MCQs relates to the development of high-quality incorrect answer options, or “distractors” as they are commonly referred in the psychometrics literature [4]. In some cases, distractors represent implausible options that weaken discriminatory power and add little value to the quality of the MCQ [5,6]. The use of non-optimal distractors (e.g., distractors selected by fewer than 5% of examinees) has been identified as a common problem shared across a wide range of undergraduate MCQs [6]. One solution to the use of non-optimal distractors would be to reduce the number of incorrect answers found in a MCQ.

The optimal number of distractors provided in a MCQ remains the subject of debate. Some educators advocate for the inclusion of five or more options in an MCQ in order to reduce the influence of chance in answering the question [7]. Previous studies have shown that 3-option MCQs perform equally well as 4-option MCQs in this regard and the use of additional options often do not improve score reliability or validity [8,9,10,11]. Other studies have also shown improved student performance and increased test taker preference for the use of 3-option MCQs [8,12]. These studies, as well as a meta-analysis of the available data, have prompted some researchers to recommend the use of 3-option MCQ items because of the difficulty in writing effective distractors [13]. Despite this evidence, many MCQs used in veterinary schools include four or more options. To date, we are unaware of any research at the classroom assessment level that has investigated the item performance of 3-option versus 4-option MCQs in veterinary medicine. The purpose of the present study was to evaluate item performance on 3-option versus 4-option MCQs used in a core curriculum course in veterinary toxicology taught at North Carolina State University (NCSU).

## 2. Materials and Methods

### 2.1. Design and Instrumentation

This study evaluated the performance of examination items administered to two classes of veterinary students enrolled in a semester-long veterinary toxicology course taught at NCSU’s veterinary college in the Spring semester of 2017. As part of a curricular redesign within the college, the course uniquely was taught twice (one for second-year students and one for third-year students) within the same semester. A quasi-experimental, crossover design was used in which students in each class were randomly assigned to take one of two versions (A or B) of a midterm (Test #1) or final exam (Test #2) [14]. Each exam version had an equal number of eighteen 3-option and eighteen 4-option MCQs. Stems used for 3-option MCQs in version A were identical to the stems used in the 4-option MCQs found in version B. The course instructor (DCD) is a veterinary toxicologist and used his expert judgment to identify the least plausible distractor to remove from each 4-option MCQ. As part of an unconventional course policy, the instructor requires students to answer only 33 of the 36 questions presented in each exam. This policy is based in part on a personal philosophy that guessing will contaminate score validity, thus allowing students to delete three items in which they likely will make a guess will help improve score integrity. As part of this process, students identify the three items they would like to treat as unscored by marking “do not grade” as the answer option.

Each mid-term and final examination administered was a required component of the course. However, for the purposes of this study, data were stripped of any identifying information by the course instructor and only the anonymous response vectors were retained. Data were then analyzed by the lead author, a professional psychometrician. The institution’s IRB declared the study ‘Exempt’.

### 2.2. Sample

The sample frame consisted of a total of 101 second-year students and 99 third-year students. With respect to demographic characteristics, 75 (74.3%) students identified as female and 26 (25.7%) as male. Of these 101 students, 7 (6.93%) identified as Black or African American, 72 (72.28%) as White/Caucasian, and 21 (20.79%) as Other. Among third-year students, 78 (78.8%) identified as female and 21 (21.2%) as male. Of these 99 students, 81 (81.82%) identified as White/Caucasian and 18 (18.28%) identified as Other.

### 2.3. Analysis

Data analysis consisted of comparing item difficulty values (*p*-values, also commonly referred to as percent correct) and the discrimination coefficient (point biserial correlation) across test forms. Because the data were mostly normally distributed, both parametric and non-parametric procedures were performed (e.g., independent samples *t*-test and Mann-Whitney *U* test) to inferentially compare values. All significance testing was performed with alpha set to 0.05. Cohen’s *d* effect size estimates were also computed to determine the practical significance of the findings. Finally, correlations (Pearson’s *r* and Spearman’s *ρ*) were calculated to measure the association between item properties across forms.

## 3. Results

For Test #1, six items were removed from scoring, thus reducing the total number of items on this exam from 36 to 30. The reasons for the deletion included a combination of concerns regarding content and psychometric functioning. For the remaining 30 items, *p*-values ranged from 0.47 to 1.00 with a mean of 0.83 (SD = 0.16) for form A. *p*-values ranged from 0.64 to 1.00 with a mean of 0.86 (SD = 0.14) for form B. An independent samples *t*-test indicated the differences were not statistically significantly different (*p* = 0.490). The non-parametric equivalent Mann-Whitney *U* test also indicated the scores were not significantly different (*p* = 0.796). The Cohen’s *d* effect size was 0.199. With respect to discrimination coefficients (DI), the mean DI value of form A items was 1.04 (SD = 0.14), and the mean DI value of form B was 1.00 (SD = 0.12). These values also were not statistically significantly different based on the *t*-test (*p* = 0.280) and the Mann-Whitney *U* test (*p* = 0.605). The Cohen’s *d* effect size was 0.306. The Pearson’s *r* correlation coefficient was 0.858 for *p*-values and 0.231 for DI values across forms, and the Spearman’s *ρ* correlation coefficient was 0.847 for *p*-values and 0.291 for DI values across forms. Results indicate the overall mean difference between each item pair was 0.03 (SD = 0.08) for *p*-values and 0.04 (SD = 0.16) for DI coefficients. The Cohen’s *d* effect size was 0.079. A complete breakdown of results is presented in Table 1.

For Test #2, *p*-values ranged from 0.64 to 1.00 with a mean of 0.89 (SD = 0.11) for form A. *p*-values ranged from 0.58 to 0.99 with a mean of 0.90 (SD = 0.11) for form B. An independent samples *t*-test indicated the differences were not statistically significantly different (*p* = 0.821). The non-parametric equivalent Mann-Whitney *U* test also indicated the scores were not statistically significant different (*p* = 0.346). The Cohen’s *d* effect size was 0.090. With respect to discrimination coefficients (DI), the mean DI value of form A items was 0.99 (SD = 0.17), and the mean DI value of form B was 0.98 (SD = 0.12). These values also were not significantly different based on the *t*-test (*p* = 0.876) and the Mann-Whitney *U* test (*p* = 0.330). The Cohen’s *d* effect size was 0.067. The Pearson’s *r* correlation coefficient was 0.927 for *p*-values and 0.583 for DI values across forms, and the Spearman’s *ρ* correlation coefficient was 0.794 for *p*-values and 0.395 for DI values across forms. Furthermore, the differences between *p*-value and DI coefficients are expressed using absolute values. The results indicate that the overall mean difference between each item pair was 0.01 (SD = 0.04) for p-values and 0.01 (SD = 0.14) for DI coefficients. The Cohen’s *d* effect size was 0.000. A complete breakdown of results is presented in Table 2.

## 4. Discussion

Results indicate that both the 3-option and 4-option MCQs resulted in similar psychometric properties. We conclude, therefore, that items containing 3-options are equally effective as items with 4-options with respect to both item difficulty and item discrimination. Our findings are in general agreement with previous studies evaluating the performance of medical [15,16,17] and nursing students [18,19] given MCQs with three to five options. These studies showed no statistically significant differences in item discrimination, item difficulty, or the mean examination scores when MCQs were administered with three versus four option answer choices [15,16,17,18]. Our data suggest that the tradition of using MCQs with four or more options in veterinary education is no longer supported by empirical evidence.

In most cases, the least plausible distractor removed from the four option MCQs were chosen by less than 5% of the students. We contend that the use of this third distractor in a 4-option MCQ was an exercise in futility in many instances, as there was no evidence of any meaningful improvements to item functioning as a result of the additional distractor. This conclusion is supported by other studies that have evaluated the impact of less than optimal distractors on MCQ performance [5,6,19]. Our data suggests that veterinary educators should consider developing MCQs with three high quality options (one correct answer and two plausible distractors). This approach could be associated with multiple benefits including reduced time needed to create MCQs. For example, three option tests and examinations are easier to write and administer and studies evaluating nurse educators have shown that nursing faculty can write more questions with three options in the same time required to write four or more options [20]. We also anticipate that veterinary students will spend less time evaluating response options when fewer distractors are presented, thereby decreasing the amount of time students spend on each test item. Since the time needed to evaluate 3-option MCQs should be shorter, educators can include additional items on an assessment to further ensure adequate content coverage, reduce the size of associated standard error estimates, increase the statistical precision of scores, and increase the reliability of scores given the well-documented relationship between increases in reliability when additional items are included [21,22].

One concern veterinary educators may have relates to the increased odds that guessing has with 3-option versus 4-option MCQs (i.e., 33% vs. 25%). This concern can be alleviated in part by using exams with a larger number of 3-option MCQs. For example, an exam consisting of 30 items would require a minimum (raw) cut score of 15 (i.e., pass rate set at 50%) if the items consisted of 3 response options in order to ensure examinees could not achieve the lowest meaningful performance category by random guessing given a 5% maximum error tolerance [23]. Another concern that veterinary educators may have with respect to our study is the generalizability of our findings, since the present study involved only one veterinary medical college located in the United States. We contend that the findings from our study, however, are consistent with those of other studies performed in other health professions settings. This convergence of findings provides evidence that supports the external aspect of validity [24].

## 5. Conclusions

In summary, this study sought to evaluate item performance on 3-option versus 4-option MCQs used in a core curriculum course in veterinary toxicology at a large veterinary medical school. Both the 3-option and 4-option MCQs resulted in similar psychometric properties. In conclusion, the findings of our study support earlier research in other medical disciplines and settings that likewise concluded there was no significant change in the psychometric properties of three option MCQs when compared to the traditional MCQs with four or more options.

## Figures and Tables

**Table 1:** Item Statistics for Test #1, Forms A and B (*N* = 30).

Item	Form A			Form B			*p*-Value Δ (Absolute)	DI Δ (Absolute)
	# Options	*p*-Value	DI	# Options	*p*-Value	DI		
1	4	1.00	1.00	3	0.96	1.02	0.04	0.02
2	4	0.94	1.05	3	0.94	0.94	0.00	0.11
3	4	0.71	1.21	3	0.74	1.17	0.04	0.04
4	4	0.92	1.01	3	0.90	0.92	0.02	0.09
5	4	0.98	1.05	3	1.00	1.00	0.02	0.05
6	4	0.93	0.96	3	0.94	1.01	0.01	0.05
7	4	0.90	0.97	3	0.95	0.98	0.04	0.01
8	4	0.79	1.00	3	0.88	0.97	0.09	0.03
9	4	0.75	0.97	3	0.87	1.04	0.12	0.07
10	4	0.91	1.02	3	0.90	1.08	0.01	0.06
11	4	0.96	1.03	3	0.98	1.00	0.02	0.03
12	4	0.53	1.19	3	0.42	0.60	0.11	0.59
13	4	0.46	1.40	3	0.79	1.08	0.33	0.32
14	4	0.92	0.93	3	1.00	1.00	0.08	0.07
15	4	0.97	0.92	3	0.99	0.99	0.02	0.07
16	4	0.60	1.18	3	0.82	1.02	0.23	0.16
17	3	0.87	0.98	4	0.90	0.96	0.03	0.02
18	3	0.91	1.05	4	0.91	1.00	0.00	0.05
19	3	0.89	0.92	4	0.92	0.98	0.03	0.06
20	3	0.93	1.07	4	0.86	1.14	0.07	0.07
21	3	0.99	1.02	4	0.95	0.88	0.04	0.14
22	3	0.87	0.97	4	0.86	1.04	0.01	0.07
23	3	0.69	1.26	4	0.67	1.35	0.02	0.09
24	3	0.94	0.95	4	0.97	1.02	0.03	0.07
25	3	0.92	0.93	4	0.96	0.99	0.03	0.06
26	3	0.99	1.07	4	0.98	0.99	0.01	0.08
27	3	0.75	1.10	4	0.75	1.17	0.00	0.07
28	3	0.47	0.75	4	0.57	0.89	0.10	0.14
29	3	0.95	1.00	4	0.90	1.07	0.05	0.07
30	3	0.56	1.43	4	0.55	0.98	0.01	0.45

**Table 2:** Item Statistics for Test 2, Forms A and B (*N* = 36).

Item	Form A			Form B			*p*-Value Δ (Absolute)	DI Δ (Absolute)
	# Options	*p*-Value	DI	# Options	*p*-Value	DI		
1	4	0.96	1.11	3	0.96	1.06	0.00	0.05
2	4	0.77	0.85	3	0.73	0.97	0.04	0.12
3	4	0.94	0.97	3	0.96	0.86	0.01	0.11
4	4	0.99	0.96	3	0.99	1.09	0.00	0.13
5	4	0.99	1.07	3	0.96	1.10	0.03	0.03
6	4	0.91	1.06	3	0.98	1.00	0.07	0.06
7	4	0.87	1.14	3	0.86	1.01	0.00	0.13
8	4	0.66	0.56	3	0.60	0.49	0.06	0.07
9	4	0.74	0.77	3	0.81	0.96	0.06	0.19
10	4	0.97	1.00	3	0.99	1.03	0.01	0.03
11	4	0.98	0.97	3	0.98	1.00	0.00	0.03
12	4	0.80	1.33	3	0.74	0.90	0.06	0.43
13	4	0.94	1.00	3	0.99	1.00	0.05	0.00
14	4	0.97	1.10	3	0.96	1.01	0.01	0.09
15	4	0.97	0.98	3	0.98	1.05	0.01	0.07
16	4	0.91	1.04	3	0.97	0.96	0.06	0.08
17	4	0.85	0.99	3	0.95	1.08	0.10	0.09
18	4	0.77	0.86	3	0.91	0.95	0.13	0.09
19	3	0.99	1.00	4	0.98	1.04	0.01	0.04
20	3	1.00	1.00	4	0.95	1.08	0.05	0.08
21	3	0.74	0.96	4	0.74	0.87	0.01	0.09
22	3	1.00	1.00	4	0.96	1.10	0.04	0.10
23	3	0.82	0.91	4	0.82	1.05	0.00	0.14
24	3	0.99	1.01	4	0.97	0.99	0.02	0.02
25	3	0.64	1.50	4	0.58	1.09	0.05	0.41
26	3	0.84	1.11	4	0.89	0.96	0.05	0.15
27	3	0.88	0.81	4	0.91	0.94	0.03	0.13
28	3	0.99	0.93	4	0.97	1.08	0.02	0.15
29	3	0.92	1.10	4	0.89	1.23	0.03	0.13
30	3	1.00	1.00	4	1.00	1.00	0.00	0.00
31	3	1.00	1.00	4	0.97	0.94	0.03	0.06
32	3	0.81	0.97	4	0.82	0.77	0.01	0.20
33	3	0.95	1.00	4	0.99	1.02	0.04	0.02
34	3	0.99	0.98	4	1.00	1.00	0.01	0.02
35	3	0.67	0.53	4	0.64	0.68	0.02	0.15
36	3	0.95	0.98	4	0.99	0.99	0.04	0.01
